# EUS-guided drainage for pelvic abscesses: a Chinese single-center two-year follow-up study highlighting clinical outcomes

**DOI:** 10.1186/s12876-026-04741-5

**Published:** 2026-05-02

**Authors:** Tao Yang, Yi Lu, Wenru Li, Jun Deng, Tao Liu, Yanan Liu, Min Zhi, Jiachen Sun

**Affiliations:** 1 Department of Gastrointestinal Endoscopy & Gastroenterology, The Sixth Affiliated Hospital, Sun Yat-Sen University, 26 Yuancun Erheng Road, Guangzhou, 510655 China; 2 Guangdong Provincial Key Laboratory of Colorectal and Pelvic Floor Diseases, The Sixth Affiliated Hospital, Sun Yat-Sen University, Guangzhou, 510655 China; 3 Biomedical Innovation Center, The Sixth Affiliated Hospital, Sun Yat-Sen University, Guangzhou, 510655 China; 4 Department of Radiology, The Sixth Affiliated Hospital, Sun Yat-Sen University, Guangzhou, 510655 China; 5 Department of Gastroenterology, The Sixth Affiliated Hospital, Sun Yat-Sen University, Guangzhou, 510655 China

**Keywords:** Endoscopic Ultrasound, Pelvic Abscess, Drainage, Crohn's Disease, Retrospective Case Series

## Abstract

**Background:**

Endoscopic ultrasound (EUS)- guided drainage has emerged as a novel technique for managing pelvic abscesses. This single-center retrospective case series aims to assess the safety and efficacy of EUS-guided drainage in treating pelvic abscesses of varying etiologies from 2021 to the present.

**Methods:**

Consecutive patients with pelvic abscesses who underwent EUS-guided drainage were retrospectively reviewed. Etiologies included appendiceal abscess secondary to acute appendicitis (*n* = 1), pelvic abscesses resulting from anastomotic leaks following rectal cancer surgery (*n* = 2), and perianal abscesses associated with Crohn’s disease (*n* = 7). The primary outcome was technical success and reduction in abscess cavity size, assessed via follow-up imaging. Clinical success was defined as significant reduction or complete resolution of the abscess cavity size on follow-up imaging at one-month post-procedure, accompanied by clinical symptom resolution and without the need for additional interventions. Secondary outcomes included post-procedural complications and resolution of the abscess without additional interventions.

**Results:**

EUS-guided drainage was technically successful in all cases. The median reduction in abscess size was statistically significant (Mean SD: 24.1 ± 11.11, *p* < 0.05). During follow-up, imaging results confirmed significant reduction in the size of pelvic abscesses in 9 patients, except for one case at the 1-month post-procedure. None of the patients required further surgical intervention, and 2 cases recurrences were observed in the sixth- and tenth-months post-procedure. Additionally, no procedure-related complications were reported.

**Conclusion:**

EUS-guided drainage is a safe and effective therapeutic option for managing pelvic abscesses of various etiologies. Its efficacy, particularly in Crohn’s disease-related cases, and the absence of complications in this cohort, suggest significant potential for broader clinical application.

**Supplementary Information:**

The online version contains supplementary material available at 10.1186/s12876-026-04741-5.

## Background

Pelvic abscesses represent a significant clinical challenge, frequently arising as complications following colorectal or gynecological surgery or in association with various conditions such as perforated viscus, Crohn’s disease (CD), appendicitis, diverticulitis, ischemic colitis, endocarditis, and sexually transmitted infections [1]. These abscesses were associated with substantial morbidity and mortality, underscoring the importance of developing effective therapeutic strategies [2].

Historically, surgical intervention has been the primary treatment modality, particularly in cases of perforation or failure of minimally invasive techniques. However, noninvasive approaches have increasingly gained acceptance as first-line treatments [3]. Among these, ultrasound-guided drainage has demonstrated high success rates, although its utility is restricted to abscesses that are accessible to an ultrasound probe. Computed tomography (CT)-guided percutaneous drainage provided an alternative for deep pelvic collections but carried limitations, including puncture site pain and the inability to place transmural stents, often necessitating the use of uncomfortable and potentially painful drainage catheters [4–6]. The technical challenges associated with the drainage of deep pelvic collections are further complicated by the presence of surrounding anatomical and vascular structures.

Endoscopic ultrasound (EUS) presents a distinct advantage in such cases, as it allowed for direct access to the abscess without traversing other organs, given that most pelvic abscesses were within the reach of an echoendoscope [7]. Over the past decade, several case series had highlighted the safety and efficacy of EUS-guided drainage for pelvic abscesses [8, 9]. However, large-scale or multicenter studies remain limited.

In this context, the present study documents a single-center experience in China, focusing on the management of pelvic abscesses through EUS-guided drainage from 2021 to the present. The study cohort comprises 10 cases of pelvic abscesses, with etiologies including appendicitis, postoperative complications of rectal cancer surgery, and Crohn’s disease-associated perianal abscesses. This investigation aims to contribute to the expanding body of evidence supporting EUS-guided drainage as a minimally invasive and effective alternative to surgery for managing pelvic abscesses, while also providing insights into its application within a Chinese population.

## Methods

This study was designed and reported in accordance with the STROBE (Strengthening the Reporting of Observational Studies in Epidemiology) guidelines to ensure transparency and completeness in reporting observational research.

This study was a retrospective, single-center case series conducted at a tertiary care center in China between January 2021 and December 2023. The study included 10 consecutive patients with pelvic abscesses who were referred for endoscopic ultrasound (EUS)-guided drainage.

### Patient selection

Inclusion criteria required that patients have intra-abdominal or pelvic abscesses located within 3 cm of the intestinal wall and measuring at least 2 cm in size. Patients were selected if they were unable to undergo effective drainage via ultrasound or computed tomography (CT) guidance. Exclusion criteria included individuals with coagulation disorders, those unable to tolerate the procedure due to cardiac or pulmonary comorbidities, individuals with gastrointestinal perforation, and those with malignant abscesses that could potentially disseminate. This selection process ensured a homogeneous patient population suitable for evaluating the efficacy of alternative drainage methods.

All patients underwent a pelvic computed tomography scan before the procedure to determine the precise size and location of the abscess. Abscesses were classified as either perirectal or pericolonic. Perirectal abscesses were defined as those located within 10 cm of the anal verge, while pericolonic abscesses were defined as those within 20 cm of the anal verge.

### Pre- and post-procedure management

Patients underwent bowel preparation with polyethylene glycol before the procedure. All patients were receiving systemic antibiotics (either ceftriaxone or metronidazole) at the time of the procedure, and these antibiotics were continued for five days post-procedure. Informed consent was obtained from all patients before undergoing EUS-guided drainage.

### Procedure

The pelvic abscess was identified using a linear-array echoendoscope (GF-UCT240-AL5; Olympus, Tokyo, Japan), which was employed to assess the abscess’s location, size, and anatomical relationship with surrounding structures. Color Doppler ultrasound was utilized to select a safe puncture site, ensuring avoidance of any intervening blood vessels.

A 19-gauge needle (ECHO-HD-19-A; Cook Ireland Limited, Limerick, Ireland) was used to puncture the abscess cavity, and the contents were aspirated using a 10-mL syringe. The aspirated material was sent for Gram staining and microbiological culture to guide appropriate antibiotic therapy.

**Fig. 1:**
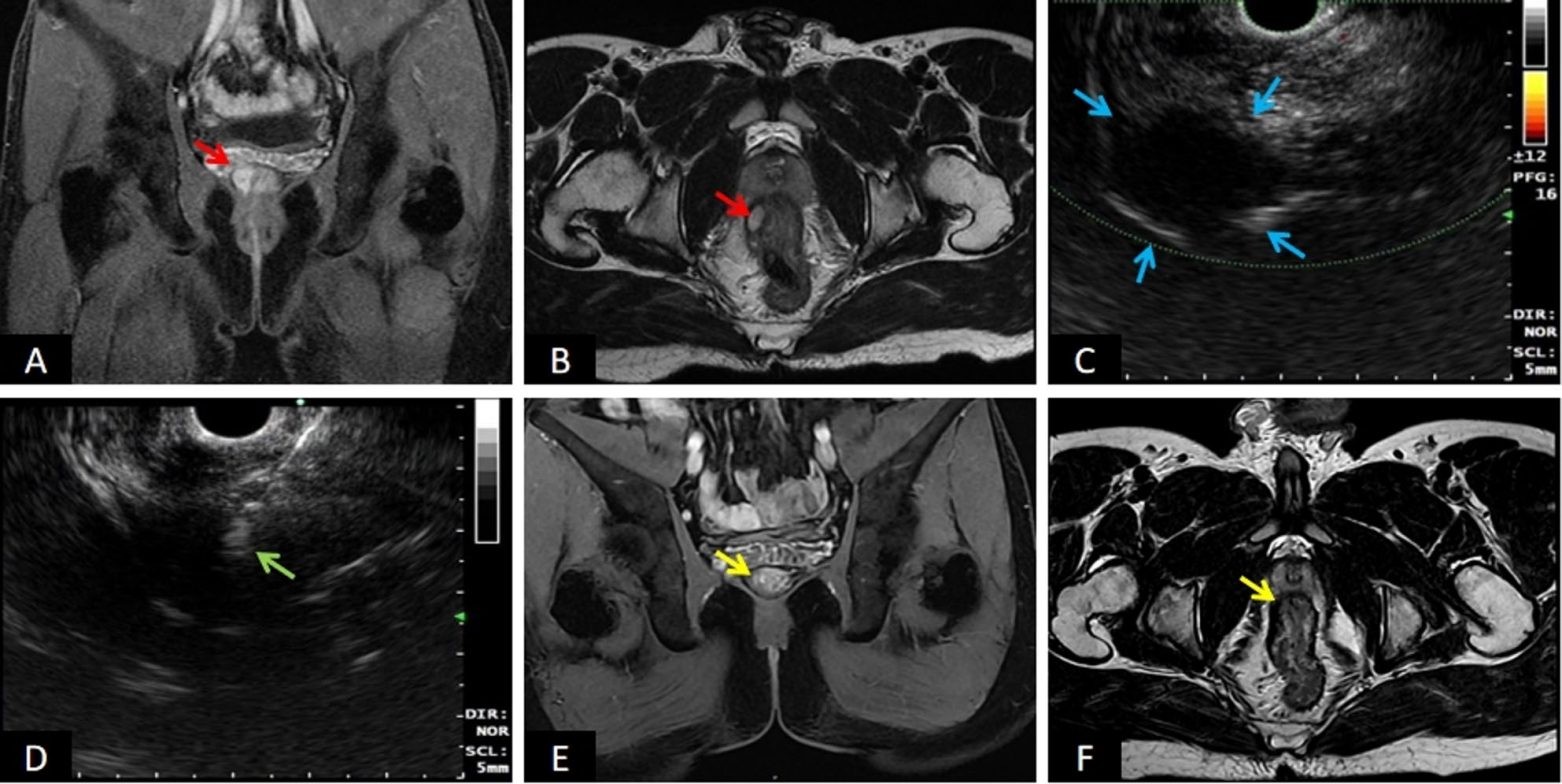
EUS-guided drainage for smaller pelvic abscesses. **A** Coronal pelvic MRI shows pelvic abscess (red arrow for abscess). **B** Transverse pelvic MRI shows pelvic abscess (red arrow for abscess). **C** EUS shows pelvic abscess (blue arrow for EUS view) and no blood vessels in the puncture approach. **D** Puncture needle is inserted into pelvic abscess under the guidance of EUS (green arrow for Puncture needle trajectory into the abscess, ). **E** Coronal pelvic MRI shows disappearance of abscess 18 months later (yellow arrow for post-procedure resolution). **F** Transverse pelvic MRI shows disappearance of abscess 18 months later (yellow arrow for post-procedure resolution)

**Fig. 2:**
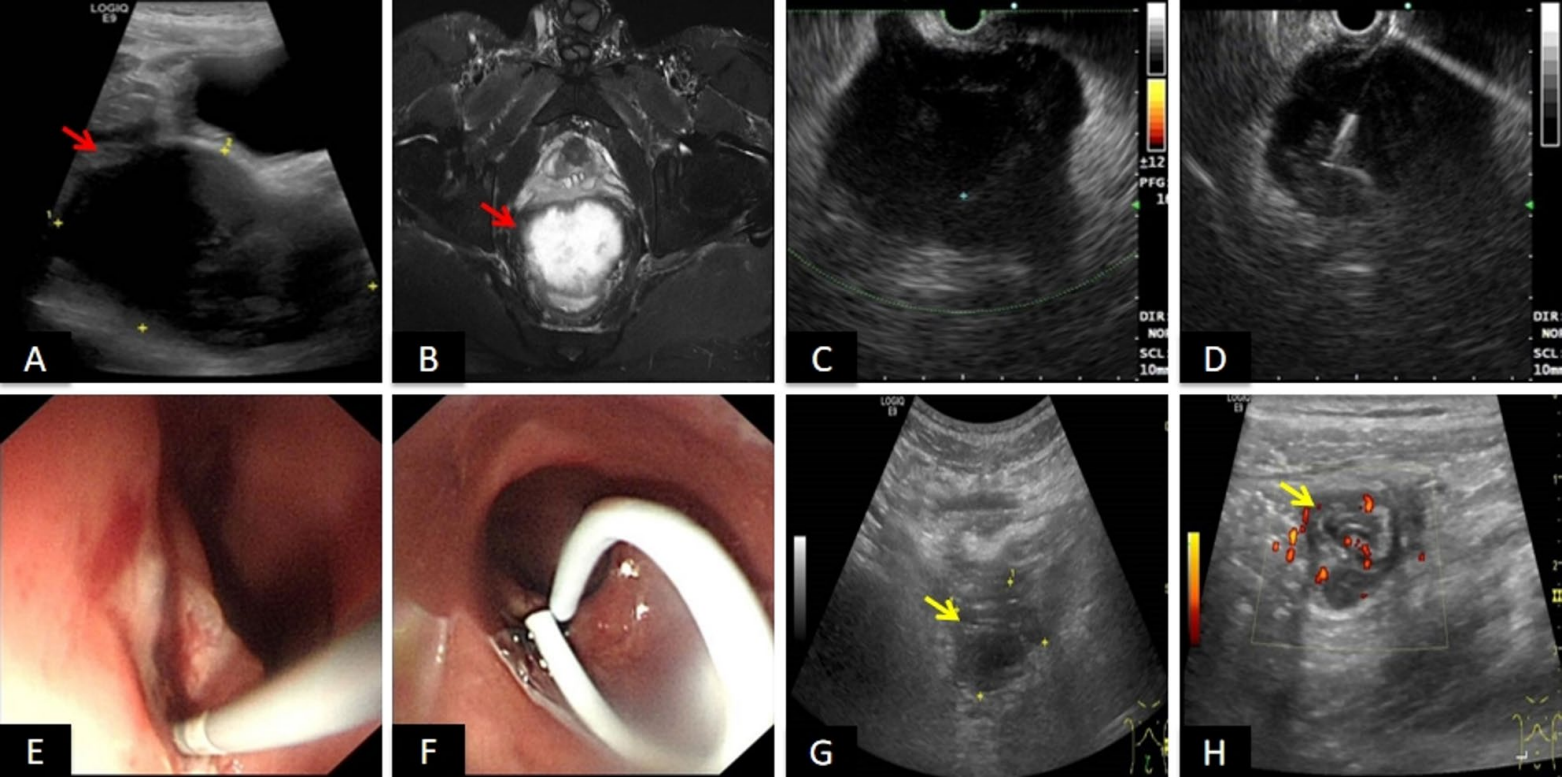
EUS-guided drainage for lager pelvic abscesses. **A** Intestinal ultrasound shows pelvic abscess (red arrow for abscess). **B** Transverse pelvic MRI shows pelvic abscess (red arrow for abscess). **C** EUS shows pelvic abscess and no blood vessels in the puncture approach. **D** Puncture needle is inserted into pelvic abscess under the guidance of EUS. **E** Fistulous tract is created by a cystotome. **F** Double-pigtail plastic stents are deployed through the fistulous tract into the abscess cavity. **G** Intestinal ultrasound shows significant reduction in pelvic abscess size one week later (yellow arrow for post-procedure resolution). **H** Intestinal ultrasound shows that pelvic abscess had almost disappeared five weeks later (yellow arrow for post-procedure resolution)

- For smaller abscesses (< 3 cm in diameter), if the initial aspiration did not yield any fluid, the abscess cavity was flushed with a metronidazole sodium chloride solution to facilitate further aspiration (Fig. 1).

- For larger abscesses (≥ 3 cm in diameter), a 0.035-inch guidewire (MO0556581; Boston Scientific Corporation, Marlborough, USA) was introduced into the cavity. This was followed by the creation of a fistulous tract using a cystotome (CST-10; Cook Ireland Limited, Limerick, Ireland).
- To ensure proper drainage, double-pigtail plastic stents (ZSS-10-3-RB; Cook Ireland Limited, Limerick, Ireland) were deployed through the fistulous tract into the abscess cavity under combined endoscopic and fluoroscopic guidance (Fig. 2). These stents were chosen as a standard, cost-effective option for drainage in this cohort.

### Post-operative and long-term follow-up

In the immediate postoperative period, all 10 patients were closely monitored for pain and signs of sepsis, including fever. Biological markers, such as C-reactive protein (CRP) levels and white blood cell (WBC) counts, were assessed within one-week post-procedure to evaluate the inflammatory response.

Each patient underwent an CT or MRI at one-month post-procedure to assess the regression of the abscess. Additionally, all patients underwent clinical follow-up to evaluate the long-term resolution of the abscess. Complications were categorized as either major or minor. Major complications included severe sepsis, perforations, or hemorrhage requiring endoscopic intervention or blood transfusion. Minor complications included fever without hypotension, self-limited bleeding, and stent migration.

Clinical success was defined as significant resolution or complete resolution the abscess cavity size on follow-up imaging at one-month post-procedure, accompanied by clinical symptom resolution (e.g., pain and fever reduction) and without the need for additional interventions.

Long-term follow-up data were obtained from medical records, clinical follow-up visits and direct communication. The follow-up period commenced on the day of EUS-guided drainage (Day 0) and concluded on the date of either a complication or recurrence necessitating surgical drainage or the last known date of patient contact. No stent-related issues (e.g., migration or clogging) requiring subsequent endoscopic intervention were observed during the follow-up period in this cohort.

### Statistical analysis

Postoperative and long-term follow-up data for the 10 patients were analyzed using SPSS Statistics software (IBM, Rochester, Minnesota, USA). Data were presented as averages, along with ranges and percentages where appropriate. For comparisons involving the reduction in abscess size, changes in CRP levels, and WBC counts, a Student’s t-test was employed. A p-value of less than 0.05 was considered statistically significant for all analyses [10].

## Results

### Patient characteristics and etiology of pelvic abscess

In this study, 10 patients with symptomatic pelvic abscesses were included. The mean age was 37.7 years, ranging from 21 to 66 years, with 70% male patients. The etiology of pelvic abscesses is detailed in Table 1, with Crohn’s disease (CD) being the most prevalent cause, accounting for 70% of cases. Other causes included appendicitis and postoperative leaks following rectal cancer surgery.

**Table 1:** Patient characteristics and causes of pelvic abscess

Patients	*n*
Age, mean (range), years	37.7(21–66)
Sex, n (%)	
Male	7(70%)
Female	3(30%)
Location of abscess	
Anterior rectal wall	6(60%)
Posterior rectal wall	4(40%)
Underlying pathology	
Appendicitis	1(10%)
Leakage after rectal cancer surgery	2(20%)
Crohn’s disease	7(70%)

### Clinical features and outcomes of EUS-guided drainage

Table 2 presents the clinical features and outcomes of patients undergoing endoscopic ultrasound (EUS)-guided drainage of pelvic abscesses.

**Table 2:** Clinical features and outcomes in patients undergoing endoscopic ultrasound (EUS)-guided drainage of pelvic abscesses

No	Abscess location	Etiology	Abscess size (mm)	Procedure	Complication	Follow-up		
						1-month post MRI/CT	Follow-up period(month)	Recurrence
1	Anterior	Appendicitis	62	Puncture, antibiotic irrigation, Stenting	no	Diminish	3	No
2	Anterior	Crohn’s disease	33	Puncture, antibiotic irrigation	no	Diminish	13	10 months Post
3	Posterior	Leaks after rectal cancer surgery	24	Puncture, antibiotic irrigation	no	Diminish	4	No
4	Anterior	Crohn’s disease	31	Puncture, antibiotic irrigation	no	Diminish	31	No
5	Anterior	Crohn’s disease	19	Puncture, antibiotic irrigation	no	Diminish	8	No
6	Posterior	Crohn’s disease	25	Puncture, antibiotic irrigation	no	Diminish	30	No
7	Anterior	Crohn’s disease	28	Puncture, antibiotic irrigation	no	Diminish	28	No
8	Anterior	Crohn’s disease	44	Puncture, antibiotic irrigation	no	Diminish	26	6 months Post
9	Posterior	Leaks after rectal cancer surgery	37	Puncture, antibiotic irrigation	no	Diminish	24	No
10	Posterior	Crohn’s disease	20	Puncture, antibiotic irrigation	no	No Diminish	16	No

Under CT or MR evaluation, the technical success rate of EUS-guided drainage was 100%. The clinical success rate at the one-month follow-up was 90%. Specifically, abscess cavity size was successfully reduced in all procedures. Except for one patient who did not decrease in size in the first month (No. 10, CD patient), no patients experienced adverse events related to the procedure, and there were no procedure-related deaths.

During a median follow-up period of 20 months (range, 3 to 31), two cases of recurrence were observed at the sixth- and tenth-months post-treatment. Both recurrent cases were patients with underlying Crohn’s disease (No. 2 and No. 8). Neither recurrence required surgical intervention and both were managed conservatively through adjustment of systemic medical therapy for CD.

### Infection marker and abscess size variations

A comprehensive analysis of infection markers and abscess size changes was conducted during the intervention. Table 3 demonstrates a significant reduction in abscess size following surgical intervention (*p* < 0.001). Although a noticeable decrease in infection markers (CRP and WBC) was observed before and after surgery, the differences were not statistically significant (*P* = 0.053. for CRP and *P* = 0.145 for WBC).

**Table 3:** Marker of infection and abscess size records during intervention periods

Size of pelvic abscess by EUS^1^	Preoperative Median (95%CI)	Postoperative Median (95%CI)	*P* ^3^
	29.5(23.02–41.57)	7.00(1.39–15.01)	<0.001*
C-reactive protein^2^	73.28(32.25-135.33)	5.98(-8.8-85.22)	0.053
White blood cell count (WBC)^2^	8.88(6.16–13.45)	7.26(6.10–8.54)	0.145

1 Records of one month before and after surgery; 2 Records of one week before and after surgery;3 Student’s t-test

*Statistically significant

## Discussion

Our study, involving a cohort of 10 patients who underwent endoscopic ultrasound (EUS) guided drainage for pelvic abscesses, adds to the existing body of evidence supporting the safety and efficacy of this minimally invasive technique. Achieving a technical success rate of 100% and a clinical success rate of 90% at the 4-week follow-up, our findings are consistent with, and in some respects exceed, those reported in the literature, including the largest series by Poincloux L et al., which documented a clinical success rate of 91.9% [11].

The technical advantages of EUS-guided drainage over alternative methods are notable. Compared to CT-guided drainage, EUS allows for the placement of internal transmural stents (double-pigtail plastic stents in this study) which eliminate the need for external, often uncomfortable, catheters and the risks associated with percutaneous puncture. Furthermore, EUS allows direct access to the abscess cavity without traversing other organs, a key benefit over surgical drainage, which is typically associated with substantial morbidity, particularly in patients with complex underlying conditions. Our findings support the use of EUS-guided drainage for pelvic abscesses secondary to medical conditions, including CD.

The observation regarding infection markers (CRP: *P* = 0.053; WBC: *P* = 0.145) is informative. While the abscess size reduction was highly significant, the post-procedure drop in systemic inflammatory markers did not reach statistical significance. This finding is potentially influenced by the high proportion of Crohn’s disease patients (70%) in our cohort. In CD, abscesses are often associated with active inflammation, and the persistence of underlying intestinal inflammation may contribute to elevated, non-significantly reduced CRP levels even after successful drainage [12, 13]. The two observed recurrences were also in CD patients, highlighting that, for this subpopulation, a successful EUS-guided drainage must be complemented by the optimization of medical therapy to control the underlying inflammatory disease activity and mitigate the heightened risk of abscess recurrence [14].

The incidence of postoperative pelvic abscesses, particularly following colorectal surgery, remains significant, with rates up to 15% as indicated by recent observational studies [15, 16]. Postsurgical colorectal abscesses accounted for 20% of cases in our study. This study illustrates that EUS-guided drainage is not only a feasible alternative to surgical intervention but also provides favorable long-term outcomes, particularly advantageous for patients who are often in poor general health and at elevated risk for surgical complications.

A significant observation in our study is the absence of major complications and the minimal nature of those that did occur, such as self-limited bleeding and stent migration, which were managed conservatively. This underscores the procedure’s overall safety and aligns with previous studies reporting low complication rates associated with EUS-guided drainage [17, 18]. Our approach with EUS-guided drainage has evolved, leading to technical refinements, based on the method described by Giovannini et al., which is efficient and straightforward [19]. Also, we utilized metronidazole sodium chloride solution instead of normal saline for flushing small abscesses, potentially enhancing infection control. For abscesses larger than 6 cm, placing an additional flushing catheter for several days was advocated to mitigate clogging and stent migration risks.

### Limitations

This study provides valuable insights into the application of EUS-guided drainage for pelvic abscesses. However, its retrospective nature, lack of comparative analysis with other drainage methods, and inherently small sample size (*n* = 10) present limitations that necessitate cautious interpretation of the results. The small cohort size specifically limits the statistical power, impacting the ability to generalize the results and to demonstrate statistical significance for markers like CRP. Future research with larger cohorts and a prospective design, including comparisons with alternative drainage techniques, is necessary to further elucidate the role of EUS-guided drainage in clinical practice.

## Conclusion

Our study demonstrates that EUS-guided drainage is a safe and effective procedure for pelvic abscesses, offering favorable long-term outcomes and serving as a viable alternative to percutaneous or surgical drainage methods. Notably, the procedure’s efficacy extends beyond postoperative complications, addressing abscesses arising from medical conditions such as Crohn’s disease. Contrary to initial concerns, our findings alleviate fears of permanent internal fistula formation, indicating that EUS-guided drainage is well-tolerated in Crohn’s disease patients without associated complications. This broadens the potential applications of EUS-guided drainage in managing pelvic abscesses, highlighting its utility in diverse clinical scenarios.

## Supplementary Information

Supplementary Material 1.

## Data Availability

Data used in this study are available from the corresponding authors upon reasonable request.

## Abbreviations

EUS: Endoscopic Ultrasound
CD: Crohn's Disease
CT: Computed Tomography
MR: Magnetic Resonance
CRP: C-reactive protein
WBC: White Blood Cell
